# How to understand high global food price? Using SHAP to interpret machine learning algorithm

**DOI:** 10.1371/journal.pone.0290120

**Published:** 2023-08-16

**Authors:** Xiao Han, Tong Yuan, Donghui Wang, Zheng Zhao, Bing Gong

**Affiliations:** Agricultural Trade Promotion Center, Ministry of Agriculture and Rural Affairs of P.R. China, Beijing, P.R. China; Monash University Malaysia, MALAYSIA

## Abstract

The global food prices have surged to historical highs, and there is no consensus on the reasons behind this round of price increases in academia. Based on theoretical analysis, this study uses monthly data from January 2000 to May 2022 and machine learning models to examine the root causes of that period’s global food price surge and global food security situation. The results show that: Firstly, the increase in the supply of US dollars and the rise in oil prices during pandemic are the two most important variables affecting food prices. The unlimited quantitative easing monetary policy of the US dollar is the primary factor driving the global food price surge, and the alternating impact of oil prices and excessive US dollar liquidity are key features of the surge. Secondly, in the context of the global food shortage, the impact of food production reduction and demand growth expectations on food prices will further increase. Thirdly, attention should be paid to potential agricultural import supply chain risks arising from international uncertainty factors such as the ongoing Russia-Ukraine conflict. The Russian-Ukrainian conflict has profoundly impacted the global agricultural supply chain, and crude oil and fertilizers have gradually become the main driving force behind the rise in food prices.

## Introduction

The global spread of COVID-19 has led to a steady increase in global commodity prices. In May 2022, the World Bank’s agricultural commodity price index reached 133.8, increasing by 60.5% in two years, and setting a historical high since record-keeping began in 1960, garnering widespread international attention. Some scholars believe that the global food crisis began in April 2020, and the risk of further deterioration has increased due to factors such as geopolitical conflicts, extreme climate change, export restrictions due to biofuels, and production expectations [1, 2]. This increase in global food prices has a significant impact on global food security and nutrition. According to data from the Food and Agriculture Organization of the United Nations’ (FAO) Global Food Crisis Report, around 193 million people in 53 countries and regions face food insecurity at crisis levels or worse, a figure that has increased by nearly 40 million people compared to the record number in 2020 [3, 4]. Global food prices have a lesser impact on high-income countries but have a greater effect on developing countries, particularly low-income countries, and may even escalate social unrest [5].

With the 21st-century turbulence and the COVID-19 pandemic, various factors have combined, exacerbating the global food crisis. Consequently, it is essential to understand the causes of the international food price surge, the importance of these factors, and why international food prices are still soaring despite a surplus in global food supply and demand. Answering these questions is crucial in ensuring global food security and providing theoretical support for global food security governance.

## Literature review

The literature on global food prices extensively examines the factors that impact them, including supply and demand, currency, oil, climate, and trade policies. Like the previous two, the food price surge during the COVID period occurred amid excess US dollar liquidity, high oil prices, and simultaneous price hikes in other commodities such as metals. The food price surge during the COVID period, like the previous two, took place amid excess US dollar liquidity, high oil prices, and simultaneous price hikes in other commodities such as metals. Notably, the academic community has not yet arrived at a consensus on the primary causative factor(s) of the preceding food crises [5–8].

Supply and demand shocks have remained the primary catalysts of food price hikes from a historical perspective [9]. Notably, in 2008, the demand for corn grew significantly due to biofuels, according to International Food Policy Research Institute (IFPRI) studies [5]. However, the global consumption of corn for industrial purposes has declined from 37.8% in 2008 to 36.4% in 2020. Consequently, although biofuel demand has not undergone drastic changes, it has not significantly impacted the equilibrium between food supply and demand in the short term. The role of increased food demand in developing areas, like Asia, in spurring food price hikes has sparked controversy. Huang et al. (2008) noted that the demand for food in developing nations has been rising steadily over the long term and cannot trigger significant market shocks in the short run, a fact corroborated by IFPRI [5, 10]. Furthermore, China and India’s determination to attain food self-sufficiency means that their food growth requirements will only have a controlled effect on the international market.

During the previous two food crises, global demand exceeded production, leading to low stocks. Timmer (2009) asserts that inadequate investment in agricultural production during the past century was a result of low food prices and contributed to reduced global food production [11]. However, this conclusion does not apply to the food price increase during the COVID period. According to FAO data, the global grain stock-to-use ratio during the COVID period is around 29.6%, which exceeds the international food security warning line. While climate and natural disasters continue to be significant factors affecting food supply, Headley & Fan (2008) posit that climate factors are not the primary cause of food price surges [12, 13]. For instance, while natural disasters like African locust plagues, Brazil’s La Niña, and the US drought have occurred since 2020, there is no convincing evidence that such events have significantly reduced global food production and stocks.

During the international food crisis, countries frequently adopted trade restrictions to safeguard their domestic markets and consumers. Several significant agricultural exporting countries, including Vietnam, imposed tariffs, quotas, and export bans on their domestic agricultural product exports during the 2008 global food crisis. Mitra et al. (2009) identified export restrictions as the primary factor behind the world food price increase during 2007–2008 [14]. In particular, the spike in rice prices was largely attributed to export restrictions imposed by rice-exporting countries [15]. Since the onset of COVID-19, countries such as Vietnam, Russia, India, and others have implemented export restrictions at various times in order to stabilize their domestic markets.

Moreover, unconventional factors, including the depreciation of the U.S. dollar and crude oil prices, have significantly impacted international food prices, as observed during previous rounds of food price increases. Since May 2020, the WTI crude oil price has risen from $30 to a record high of $130, resulting in an increase in agricultural production, trade costs, and ultimately food prices. Recently, the correlation between crude oil prices and food prices has become even more pronounced [16, 17].

Abbott et al. (2008) identified the depreciation of the U.S. dollar and futures market speculation as crucial drivers for the general rise in agricultural and other commodities’ prices [18]. From a macroeconomic perspective, food price surge during the COVID period has been accompanied by a general increase in global commodity prices, yet the increase in food prices has been relatively small compared to other commodities. Scholars believe that the excess liquidity of the U.S. dollar is the primary cause of the upsurge during pandemic in food prices due to the quantitative easing monetary policy initiated by the United States after the global spread of COVID-19, which caused the M2 growth rate to reach a historical high [19].

Historically, the increase in international food prices has been attributed to several factors, including an increase in demand for biofuels, market speculation, trade restrictions, the increase in money supply, the rise in demand from developing nations, climate change, and natural disasters leading to food production reduction. Nevertheless, the relative significance of these variables in affecting prices has been extensively debated across different stages in the literature [17]. Furthermore, research on this recent round of food price increases is primarily qualitative [20], with a scarcity of quantitative research.

Given the complexity in analyzing the relative importance of factors affecting the surge in global food prices based on historical experience and the current situation, this paper proposes to employ a machine learning research method. Traditional econometric methods are limited in their ability to handle large datasets with numerous variables, making machine learning a suitable alternative. Previous studies on food price influencing factors that utilize machine learning algorithms are scarce [24], despite the advantages of techniques such as random forests and support vector machines in economics research [21–28]. Finally, we will draw the main conclusions and provide relevant policy recommendations.

## Theoretical framework

The analysis of factors influencing grain prices typically encompasses aspects such as grain production, consumption, and macroeconomic factors like money supply and oil prices. This paper will undertake a theoretical analysis from these perspectives.

### Supply and demand

From an economic standpoint, the analysis of grain prices can be categorized into two levels: general and specific. There are notable distinctions between general research on food crops and specific research on individual crops. Factors that may have little significance in explaining individual prices can become crucial in explaining aggregate price movements if they are common. On the other hand, substitution between crops is deemed important at the individual crop price level, but it has a lesser impact at the aggregate level. The substitution relationship between crop varieties is a crucial factor affecting the price of that particular variety when studying individual varieties. Nevertheless, when studying overall food prices or grain prices, ‘common factors’ that affect both supply and demand, have a greater influence on overall grain prices.

According to supply and demand theory, grain prices are jointly determined by the supply and demand curves. Scholars believe that in the macroeconomic reality, supply shocks tend to have a more significant impact on the equilibrium prices compared to changes in demand, which have a relatively smaller effect [17]. However, the situation is exactly the opposite for agricultural products, there is relatively little interdependence between the production of different crops, and consumers can easily choose other substitutes. As a result, demand shocks tend to have a more significant impact on the equilibrium prices compared to changes in supply.

**Fig 1:**
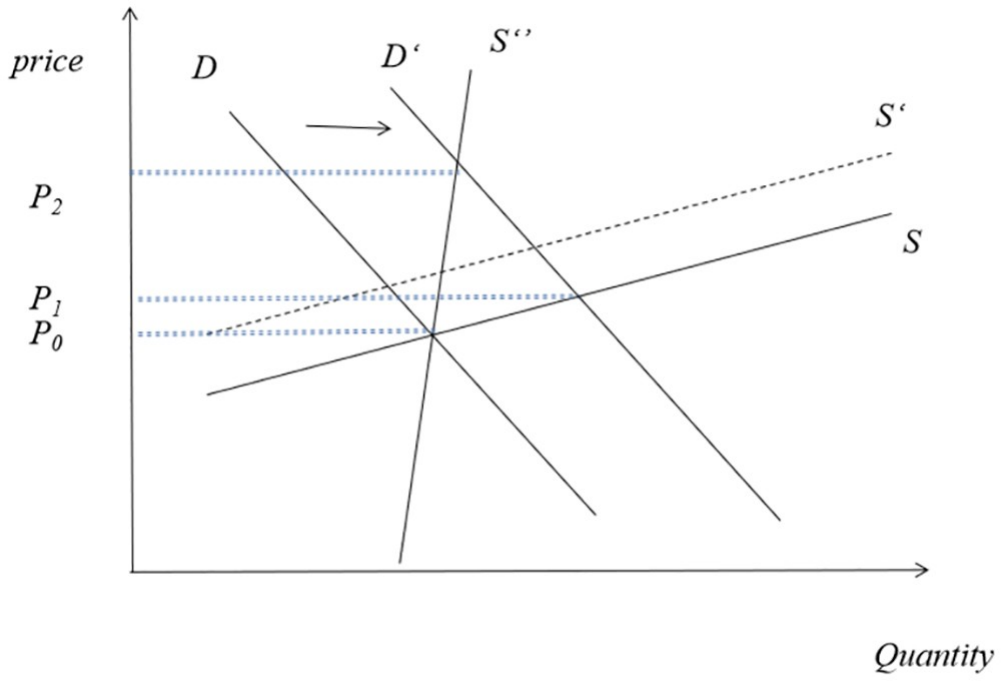
Global food price formation mechanism.

Even at the variety-specific level, demand shocks have a greater impact on equilibrium prices than supply shocks. This is because there are substitutions and correlations among agricultural products, and the supply elasticity of specific crops is relatively large. As shown in Fig 1, when demand shocks occur, they often cause complex reactions from both supply and demand sides. Changes in demand may lead to an increase in the price of intermediate goods, which in turn may lead to a synchronous increase in the price of final products. It is precisely this linkage reaction that leads to a change in the elasticity of the supply curve, from *S*′ to *S*′′. In other words, theoretically speaking, macroeconomic variables that affect demand are the main factors affecting equilibrium prices. It should be pointed out that in recent years, the impact of climate factors on supply has become increasingly significant. Climate, as a “common factor” affecting supply curves, mainly affects equilibrium prices by affecting the supply side.

### Oil prices and biofuels

**Fig 2:**
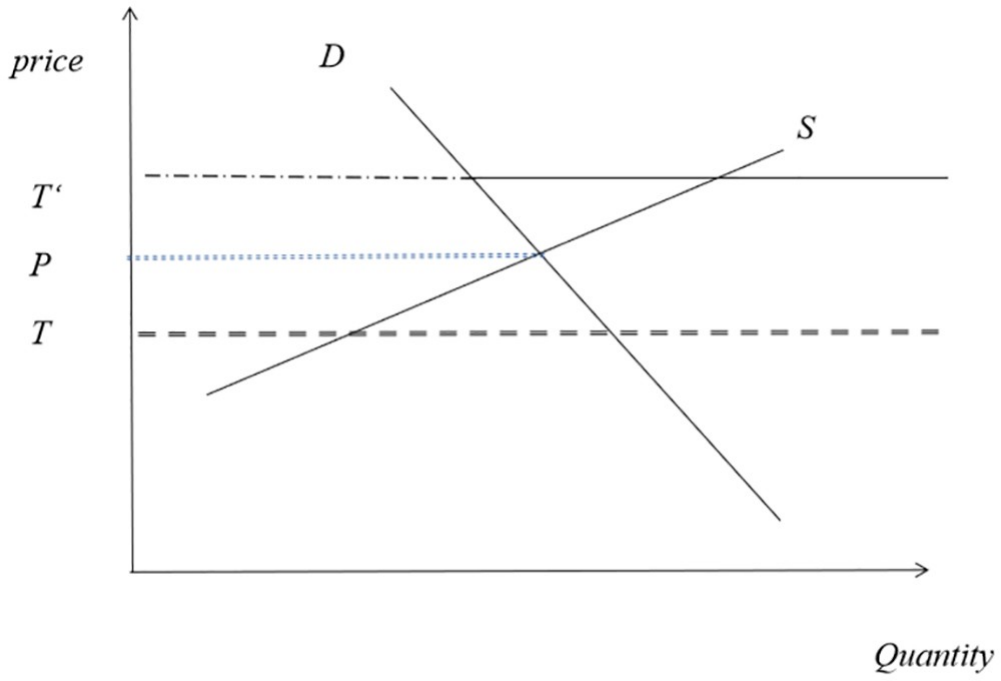
Demand for biofuels and food prices.

In recent years, there has been an increasing linkage between oil prices and grain prices. According to studies, from 2000 to 2011, the impact of oil on agricultural product prices has become increasingly significant [29]. The impact of oil prices on grain prices mainly occurs through two channels. Firstly, oil is a major component of production materials and transportation costs for agricultural products. Mitchell’s (2008) research shows that rising energy prices and freight rates will significantly increase production costs [30]. An increase in production costs means that the grain supply curve will shift upward. According to Baffes’ (2007) research, a 17% increase in oil prices will be transmitted to the agricultural product price market [31]. Secondly, oil prices also have an impact on grain demand. Roberts and Schlenker’s (2010) research found that 20%-30% of the global grain price increase in 2008 was due to increased demand for biofuels [32]. According to the analysis framework provided by Schmidhuber (2006) for biofuels, corn, and oil, as shown in Fig 2, when the oil price is below the supply-demand equilibrium point of corn, located at *T*, the relatively high price of corn makes the market lack the motivation to produce more biofuels. Conversely, when the oil price is relatively high, located at *T*′, the relatively low price of corn induces the market to produce more biofuels to replace oil [33].

### Money supply

According to classical monetary economics theory, Fisher (1911) proposed the Fisher equation, as shown in Eq 1, which explains the relationship between the quantity of money and the price of goods.

$$\begin{array}{c} MV=PT \end{array}$$ (1)

In Eq 1, *M* is the money supply; *V* is the velocity of money circulation; *P* is the price of agricultural products; *T* is the quantity of goods traded. Therefore:

$$\begin{array}{c} P=MV/T \end{array}$$ (2)

From the Fisher identity in Eq 2, it can be seen that *P* depends on *M*, *V*, and *T*. *V* is determined by the global monetary system and does not change in the short term, thus it is considered to be constant. The quantity of agricultural products traded, *T* is determined by global tradable agricultural products, and in reality, it is generally stable. Therefore, the change in the price of agricultural products, *P* will depend on the money supply *M*.

## Model selection and data description

### Machine learning algorithms

In machine learning, ensemble algorithms can be categorized into two types based on the dependencies among the classifiers: Bagging and Boosting. Random Forest belongs to the Bagging algorithm, while eXtreme Gradient Boosting (XGBoost) and categorical boosting (CatBoost) algorithms belong to the Boosting algorithms. In this paper, we plan to compare various machine learning algorithms and select the optimal algorithm for empirical estimation. Due to space limitations, this paper only briefly introduces the three main algorithms involved.

Random Forest Algorithm: Breiman (2001) first proposed the Random Forest algorithm, which is an ensemble learning algorithm based on decision trees. It is a supervised learning method that combines multiple decision trees to improve prediction accuracy. The basic principle is to construct multiple decision trees and then obtain a comprehensive result through weighting. Compared with decision trees, Random Forest has randomness in sample selection and variable selection, which can avoid the prediction differences and overfitting problems of individual decision trees. The specific mathematical formula is as follows:

$$\begin{array}{c} Y_{t}=\frac{1}{T}\sum_{h=1}^{T}l_{k}\left(x\right) \end{array}$$ (3)

The training algorithm for Random Forests(as shown in Eq 3) applies the general technique of bootstrap aggregating, or bagging, to tree learners. Given a training set *X* with responses *Y*, bagging repeatedly (*T* times) selects a random sample with replacement of the training set and fits trees to these samples. After training, predictions for unseen samples *x* can be made by averaging the predictions from all the individual regression trees on *x*. *l*_*k*_(.) represents the set of the *K*_*th*_ random tree.

XGBoost Algorithm: XGBoost was proposed by Chen and Guestrin (2016) and is an improvement of the Gradient Boosting Decision Tree (GBDT) algorithm. It uses normalization in the objective function to reduce complexity and prevent overfitting. XGBoost is also an ensemble model composed of decision trees, and its prediction performance is better than that of a single decision tree model. The specific mathematical formula is:

$$\begin{array}{c} Y_{i}^{T}=\sum_{h=1}^{T}\;f_{k}\left(x_{i}\right)=y_{i}^{T-1}+f_{T}\left(x_{i}\right) \end{array}$$ (4)

In Eq 4, $y_{i}^{T-1}$ is the generated tree, and *f*_*T*_(*x*_*i*_) is the new generated tree model. *T* is the number of all tree models. CatBoost Algorithm: CatBoost algorithm is a GBDT framework based on oblivious trees as basic learners, with few parameters, support for categorical variables, and high accuracy. Compared with XGBoost, it can efficiently and reasonably handle categorical features by innovatively transforming categorical features into numerical features. For details, see Dorogush (2018) [34]. Variable Importance Analysis: Random Forest, XGBoost, and CatBoost algorithms are “black box” models, and measuring variable importance is particularly important. Lundberg and Lee (2017) proposed the Shapley Additive explanations (SHAP) method to interpret the results of complex models such as machine learning [35]. For each predicted sample, the model generates a predicted value, and the SHAP value is the value assigned to each variable in the sample. The specific calculation idea is to calculate the marginal contribution when a certain variable is added to the model. For this variable, there are different marginal contributions in all samples, and the mean value is the SHAP value of this variable. The calculation formula is as follows:

$$\begin{array}{c} \hat{\varphi }=\frac{1}{K}\sum_{k=1}^{K}\left(\hat{g}\left(x_{+j}^{m}\right)\right)-\left(\hat{g}\left(x_{-j}^{m}\right)\right) \end{array}$$ (5)

In Eq 5, $\hat{g}\left(x^{m}\right)$ represents the predicted function of the input sample feature *X*. In addition, Breiman (2002) also proposed that when estimating Random Forest, by randomly changing the variable sequence, observing the change in the mean square error(MSE), it can be used to evaluate variable importance. After randomly permuting the sequence of variable *X* and calculating the change in the out-of-bag error (OOB) estimation value before and after adding noise, this method can be used to evaluate the importance of all variables. If the variable *X* does not significantly affect *Y*, there is no significant difference between the two OOB mean square errors. If the model accuracy drops significantly, the variable *X* has greater importance.

### Data description

The article selected nine key variables that affect global food prices based on the theoretical analysis in the previous section and Christopher’s (2010) variable selection method. These variables are related to supply and demand, currency, crude oil, and trade policy. To measure the supply and demand aspect, the article uses the changes in grain production, consumption, and inventory published monthly by the US Department of Agriculture (USDA) as indicators, following the processing methods of Michael (2012) [36]. It should be noted that climate factors mainly affect prices through supply and can be approximately considered as included in the USDA’s forecast changes.

Additionally, the article uses global grain consumption variables to indirectly verify whether demand from developing countries is the main factor driving the rise in global food prices. Developed countries’ total food consumption is relatively stable, while developing countries are the main driver of consumption growth [4]. Therefore, the growth impact of global food consumption demand can be approximately attributed to developing countries.

Regarding currency, the article selects a broad measure of money supply of US, M2, as a proxy indicator for global currency supply, following the classical monetary theory (Fisher equation), which states that the quantity of money is directly proportional to commodity prices. Since the COVID-19 pandemic, countries worldwide have significantly increased their money supply to stimulate their economies, and the US dollar, as a global currency, affects global currency liquidity. The article measures global crude oil prices using the West Texas Intermediate (WTI) crude oil price, which can create substitution relationships with biofuels and agricultural products and increase production costs through agricultural inputs and shipping. The global fertilizer price (Fertilizer) is selected as an indicator of intermediate cost factors.

Regarding trade policy, the article uses the Global Economic Policy Uncertainty Index (GEPU) to measure trade policy changes, following Frimpong’s (2021) research method [37]. Due to data availability, all variables in this article use data from January 2000 to May 2022 (see Table 1). Food price data (Food_price) comes from the IMF database (https://data.imf.org/). Data on global fertilizer prices (Fertilizer), US WTI crude oil futures prices (WTI_crude_oil), US dollar supply (US_M2), and US Consumer Price Index (US_CPI) come from the Wind database(https://www.wind.com.cn/). The GEPU data come from the Economic Policy Uncertainty database(https://www.policyuncertainty.com/global_monthly.html). Data on global grain production, consumption, and inventory come from the USDA database(https://apps.fas.usda.gov/psdonline/app/index.html#/app/advQuery).

**Table 1:** Descriptive statistics of sample data.

variable	mean	std	min	25%	50%	75%	max
US_M2	9.14	0.41	8.45	8.79	9.10	9.48	10.00
Fertilizer	4.72	0.45	3.83	4.47	4.71	5.03	5.81
Food_index	4.49	0.30	3.92	4.21	4.56	4.76	5.07
WTI_crude_oil	4.91	0.44	3.90	4.60	4.95	5.25	5.82
grain_production	7.71	0.14	7.50	7.59	7.71	7.84	7.94
grain_consumption	7.72	0.13	7.53	7.60	7.72	7.83	7.94
grain_stock	6.15	0.31	5.69	5.90	6.11	6.38	6.77
stock-to-use ratio	0.21	0.04	0.14	0.18	0.20	0.24	0.32
GEPU	4.82	0.48	3.89	4.47	4.78	5.13	6.06
US_CPI	2.33	1.54	-2.10	1.50	2.10	3.20	8.50
Observations:268							

### Data preprocess

This study has been divided into training (70%) and test (30%) samples in order to compare the performances of diferent machine learning models. To enhance the identification of linear relationships between the price and other variables, the logarithmic transformation has been applied to time series variables, which has been proven to be effective in enhancing the model’s fitting performance and reducing its error [38]. Following the processing methods of Sami et al.(2021) [38], we randomly partition the dataset by selecting 70% of the data as the training data set and the remaining 30% as the testing set. Table 2 gives more information about data prepressing in this study.

**Table 2:** Descriptive statistics of sample data.

Description	Value
Target	Food_index
Original Data	(268, 10)
Missing Values	False
Numeric Features	9
Categorical Features	0
Ordinal Features	False
High Cardinality Features	False
High Cardinality Method	None
Transformed Train Set	(187, 9)
Transformed Test Set	(81, 9)
Shuffle Train-Test	True

## Results

This study first estimated the data based on the full sample from January 2000 to May 2022, and then used SHAP values to analyze the data from the past two years. Thirty percent of the time series data was selected as the test set. This study experimented with sixteen machine learning algorithms using the full sample data and selected the model with the highest *R*^2^ value for parameter tuning and analysis of its results. Based on the empirical results, this study selected the CatBoost model for empirical analysis, and the mean residual of the model was 0, and the upper and lower distributions were uniform, indicating that the model had good predictive results (as shown in Table 3). It is worth noting that in order to prevent the problem of multicollinearity from affecting the interpretability of the variables, this study conducted a correlation analysis on nine variables and found that there was no multicollinearity among the variables overall. The two independent variables with high similarity are inventory consumption ratio and inventory volume, but both variables represent inventory information and do not affect the research conclusions.

**Table 3:** Machine learning algorithms selection.

Model	MAE	MSE	RMSE	R2	RMSLE	MAPE
CatBoost Regressor	**0.0243**	**0.0012**	**0.033**	**0.9854**	**0.0059**	0.0054
Extra Trees Regressor	0.0233	0.0013	0.0337	0.9839	0.006	**0.0051**
Gradient Boosting Regressor	0.0272	0.0016	0.0382	0.9807	0.0068	0.006
Light Gradient Boosting Machine	0.0322	0.0021	0.0441	0.9746	0.0079	0.0071
Random Forest Regressor	0.0296	0.0021	0.0437	0.9734	0.0078	0.0065
Extreme Gradient Boosting	0.0327	0.0024	0.046	0.9716	0.0084	0.0073
AdaBoost Regressor	0.0384	0.0028	0.0516	0.9643	0.0092	0.0084
Decision Tree Regressor	0.0358	0.0029	0.0525	0.9621	0.0094	0.0079
K Neighbors Regressor	0.0456	0.0047	0.0658	0.938	0.0118	0.0101
Bayesian Ridge	0.0626	0.0061	0.0769	0.9172	0.0140	0.0140
Ridge Regression	0.0633	0.0062	0.0769	0.9165	0.0140	0.0141
Huber Regressor	0.0629	0.0064	0.0783	0.9142	0.0143	0.0141
Linear Regression	0.0641	0.0064	0.0787	0.9135	0.0144	0.0144
Passive Aggressive Regressor	0.0769	0.0092	0.0942	0.8786	0.0170	0.0170
Orthogonal Matching Pursuit	0.1012	0.0166	0.1274	0.7688	0.023	0.0225
Lasso Regression	0.2470	0.0893	0.2962	-0.0757	0.0547	0.0563

**Fig 3:**
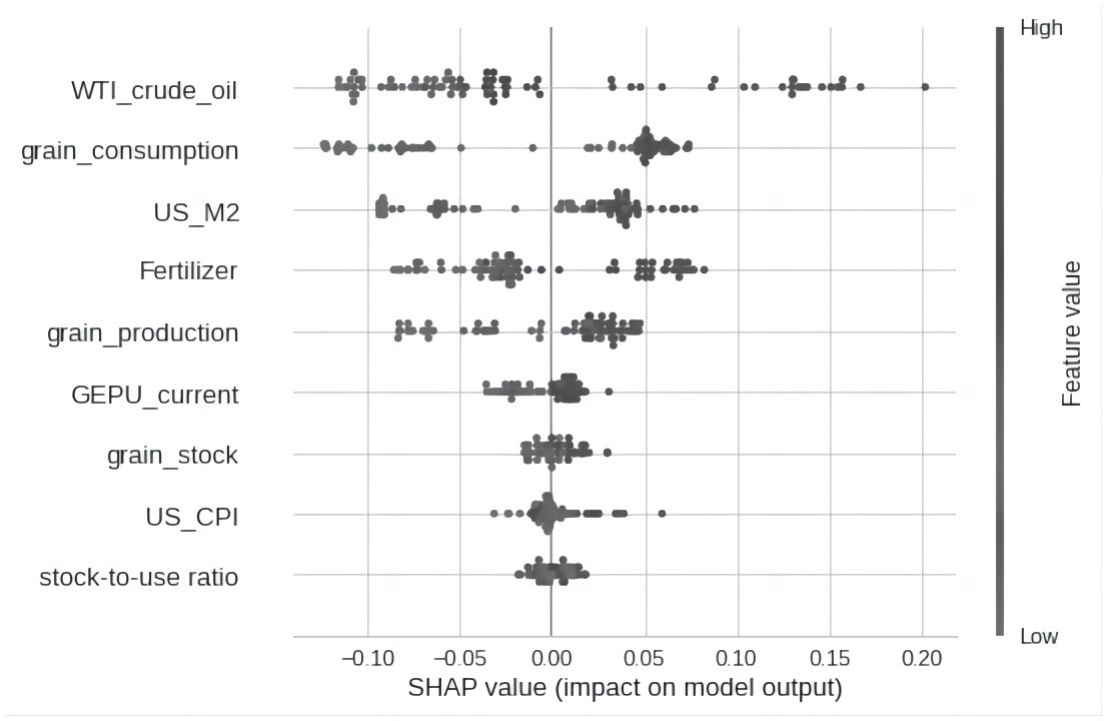
Feature importances.

**Fig 4:**
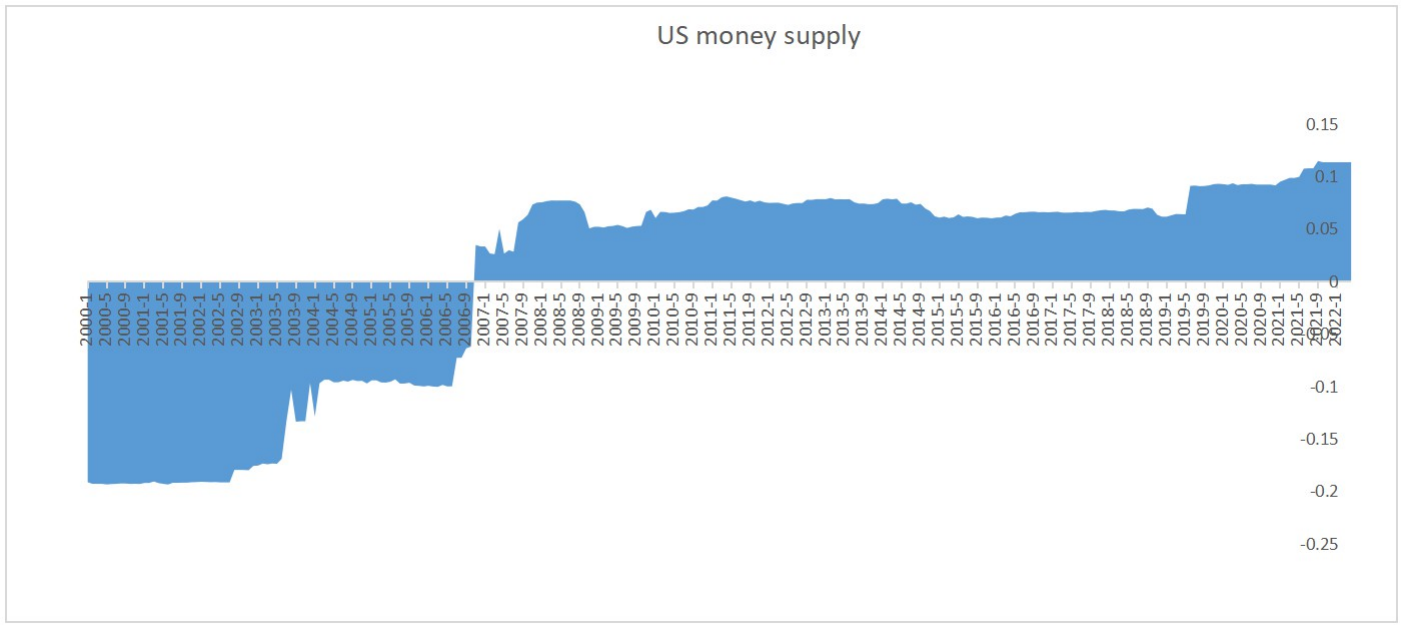
Contribution of US dollar supply to the impact of global food prices.

**Fig 5:**
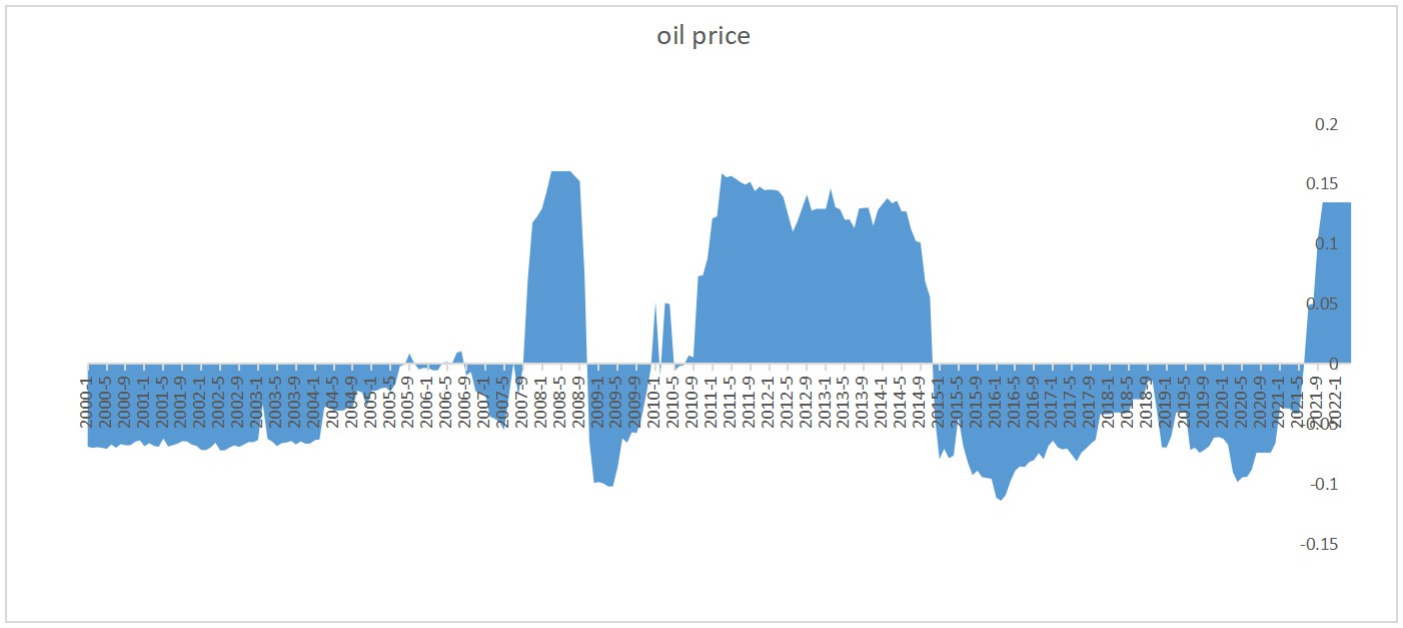
Contribution of crude oil to the impact of global food prices.

The results of the relative importance estimation of variables show that, in the full sample estimation, the three most important variables affecting grain prices since 2000 are crude oil prices, increased demand for grain consumption, and the supply of the US dollar (as shown in Fig 3), while in the past two years, crude oil and US dollar supply have been the main factors affecting grain prices (as shown in Figs 4 and 5).

Regarding crude oil, the empirical results show that extremely high oil prices (dark black dots) have a significant impact on grain prices, while low oil prices (light black dots) have a relatively neutral effect on grain prices. According to the SHAP value graph of the feature variables (as shown in Figs 4 and 5), oil crises and global food crises often occur simultaneously, such as in 2007–2008, 2011–2013, and the price increases during the COVID period, where crude oil prices have always been the main factor leading to high grain prices.

The results indicate that the impact of crude oil on the global increase of grain prices can be divided into two stages since 2020. Before July 2021, even though crude oil prices increased, they remained at historical average levels and did not have a significant impact on grain prices. After July 2021, crude oil prices surpassed a threshold, particularly after the Russia-Ukraine conflict, leading to a substantial increase in their impact on grain prices. The observation is consistent with the theoretical expectation that crude oil prices simultaneously influence supply and demand curves as well as the equilibrium price. On the supply side, downstream inputs influenced by crude oil prices, such as fertilizers, affect grain prices. On the demand side, the large-scale production of biofuels links crude oil and agricultural products together, causing a significant substitution effect. In 2021, United States Department of Agriculture (USDA) data show that approximately 130 million tons of corn for biofuel production is produced in the US, which accounts for 35% of the total corn production, equivalent to 73% of the global corn trade volume. Although corn’s industrial use comprises a relatively large proportion, the total demand has maintained stability at roughly 170 million tons. In the short term, the demand for biofuels has not significantly impacted the supply and demand of corn, and hence, it is not the dominant factor during pandemic global increase in grain prices.

Consistent with theoretical analysis, crude oil prices simultaneously affect the supply and demand curves and the equilibrium price. On the supply side, downstream inputs, such as fertilizers, are mainly affected by crude oil’s prices, which are then transmitted to grain prices. On the demand side, the large-scale production of biofuels connects crude oil and agricultural products together, and the substitution effect between the two is significantly enhanced. According to USDA data, in 2021, the US produced about 130 million tons of corn for biofuel production, accounting for 35% of the total corn production in the US, equivalent to 73% of the total global corn trade volume. Although corn’s industrial use accounts for a high proportion, its total demand has remained stable at around 170 million tons. In the short term, the demand for biofuels has not had a significant impact on the supply and demand of corn, and therefore is not the dominant factor that contributed to global increase in grain prices during the COVID period.

Although crude oil has always been one of the main variables affecting grain prices, the transmission path of crude oil’s impact on global grain prices has changed. Admittedly, the development of biofuels is an important aspect affecting food prices. However, the main reason for the increase in grain prices during pandemic is the increase in intermediate costs caused by crude oil, including the increase in the cost of agricultural materials such as fertilizers, agricultural machinery, and the cost of grain transportation by sea and land. For example, Sea transportation costs, including crude oil as a significant component, have almost tripled since the onset of the COVID-19 pandemic. According to international shipping data, shipping costs account for 11% of the total taxable cost of soybean imports into China and 20% of the total cost of corn. The shortage of shipping capacity, combined with the short-term rise in oil prices, has pushed up intermediate costs and thus driven global grain prices higher. Therefore, from the corn and biofuel markets, it can be inferred that the main impact path of crude oil on grain prices from 2010 till now should be the supply side, that is, affecting global grain prices through intermediate costs.

Regarding the supply of the US dollar, the empirical results show that the impact of the US dollar supply on the global increase in grain prices was not significant before 2008. After 2008, the impact of the US dollar supply on grain prices has significantly increased, especially since 2020 when the US implemented an unlimited quantitative easing monetary policy, causing almost all commodities priced in US dollars to rise sharply.

**Fig 6:**
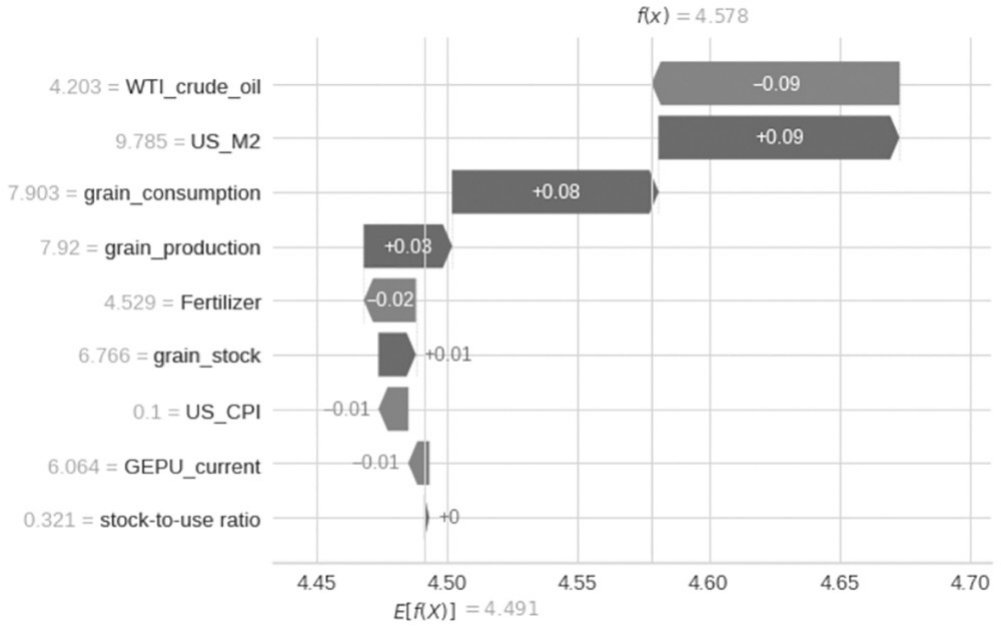
Changes in the contribution of various factors to food prices in in May 2020.

**Fig 7:**
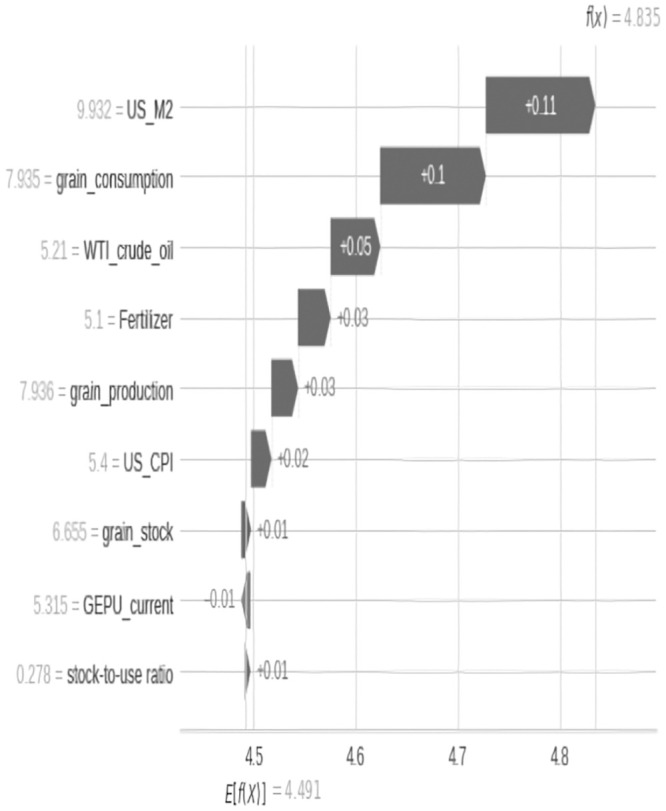
Changes in the contribution of various factors to food prices in May 2021.

**Fig 8:**
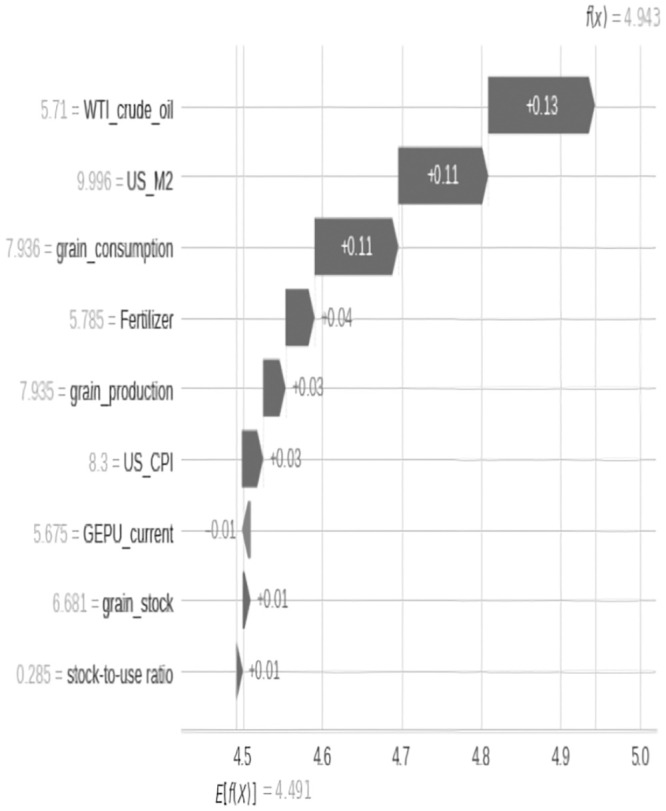
Changes in the contribution of various factors to food prices in May 2022.

From the perspective of supply and demand, changes in the structure of the grain market play an important role in the surge in grain prices. Grain prices are the result of the joint action of supply and demand (as shown in Figs 6–8). In the short term, the elasticity of supply and demand for agricultural products is very small. According to USDA reports, nearly three-quarters of the world’s energy comes from corn, soybeans, wheat, and rice, and their demand elasticity is only -0.04, while their short-term supply elasticity is 0.0–0.3. This means that even small changes in supply and demand can cause huge price changes. It is worth noting that supply and demand elasticity is not fixed, and when the supply tightens, the demand elasticity of agricultural product prices will increase rapidly, leading to a “non-linear” feature of price increases, that is, as the market supply gradually tightens, the elasticity of prices to demand will increase rapidly. Specifically, when the international market supply of agricultural products is abundant, if the demand for agricultural products increases by 1%, the price will only increase by 1%, while when the supply is in a tight balance, a 1% increase in demand may bring several times the price increase.

In addition to the factors mentioned above, other factors such as economic policy uncertainty also have an impact on global food prices. Since the outbreak of COVID-19, global economic policy uncertainty has significantly increased [39], especially since the Russia-Ukraine conflict, the increase in policy uncertainty factors such as sanctions against Russia and trade restrictions has had a comprehensive impact on global agriculture. Consistent with the research results of Frimpong (2021), global economic policy uncertainty has an increasingly significant impact on global food prices, especially since the Russia-Ukraine conflict, where the effect of fertilizer prices on grain prices is more significant than inventory variables [37].

Based on the above empirical analysis, it can be concluded that in the past two years, the US dollar money supply and crude oil prices are the two most important variables affecting grain prices. The unlimited quantitative easing monetary policy of the US dollar is the primary factor behind global increase in food prices during pandemic, and its impact is still ongoing. The main path of crude oil’s impact on this round of grain prices is cost-driven, namely the increase in intermediate costs caused by crude oil, especially the cost of transportation. The substitution effect between crude oil and biofuels has a relatively small impact on this round of grain price increases. The global supply and demand shock is another important factor affecting the rise in grain prices. Against the backdrop of a global food shortage, the complex international situation has amplified expectations of reduced crop yields and high demand, further exacerbating the global food crisis.

## Discussion on global food security risk during pandemic

Since the outbreak of the COVID-19 pandemic, global food prices have been on the rise for two years. This phenomenon can be predominantly attributed to five factors: weak supply expectations, excess liquidity of US dollars, extreme weather, export bans, and the energyization of food. The elevation of food prices has been influenced by a variety of factors throughout this period. As the pandemic first emerged, the primary driving force behind the growth in prices was the excess liquidity of US dollars, while the link between the food and energy markets intensified after the Russia-Ukraine conflict.

### Uneven distribution and increasing hunger

**Fig 9:**
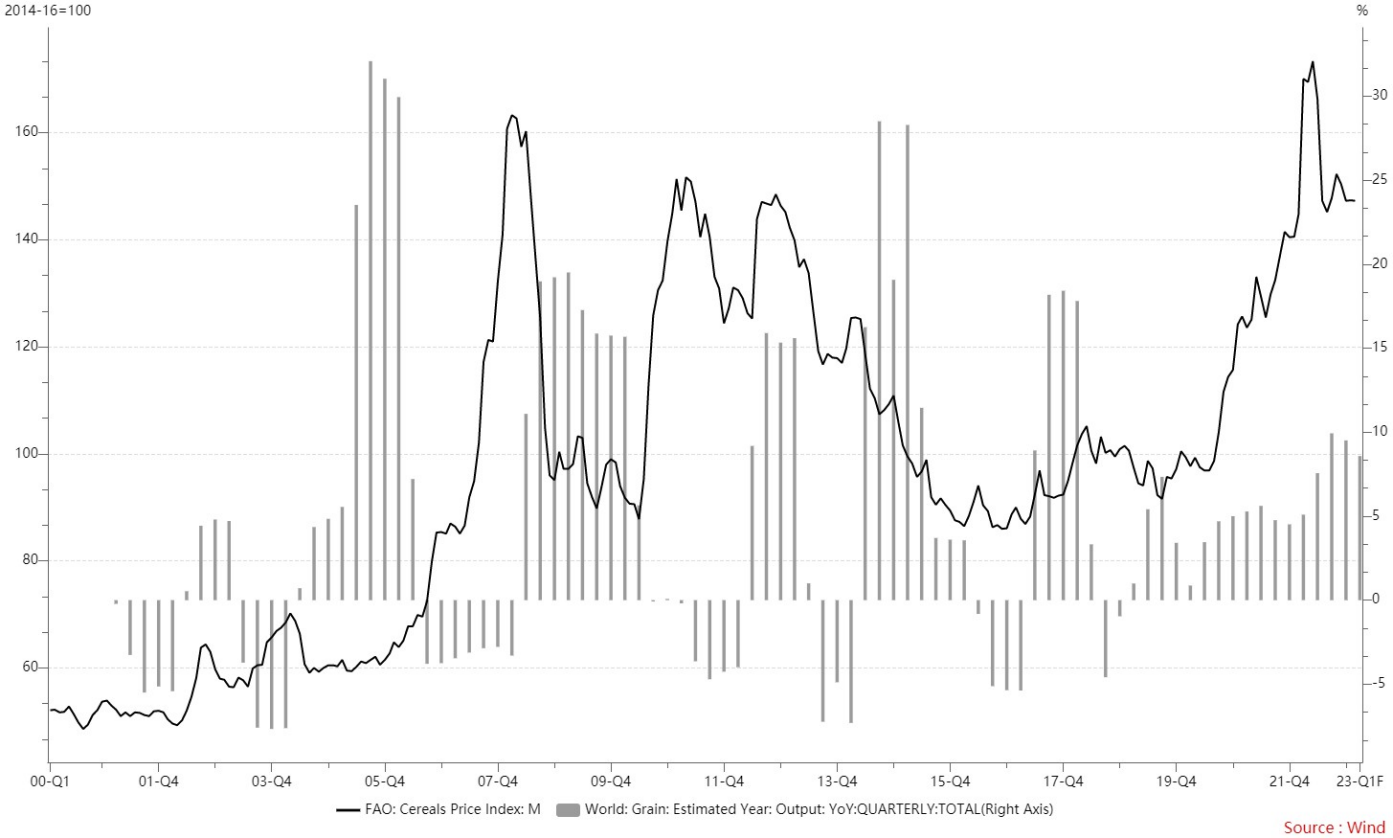
Global grain price rise and production change. The curve represents global food prices, which correspond to the vertical axis on the left-hand side. The bar represents year-over-year changes in global grain production, which correspond to the vertical axis on the right-hand side.

**Fig 10:**
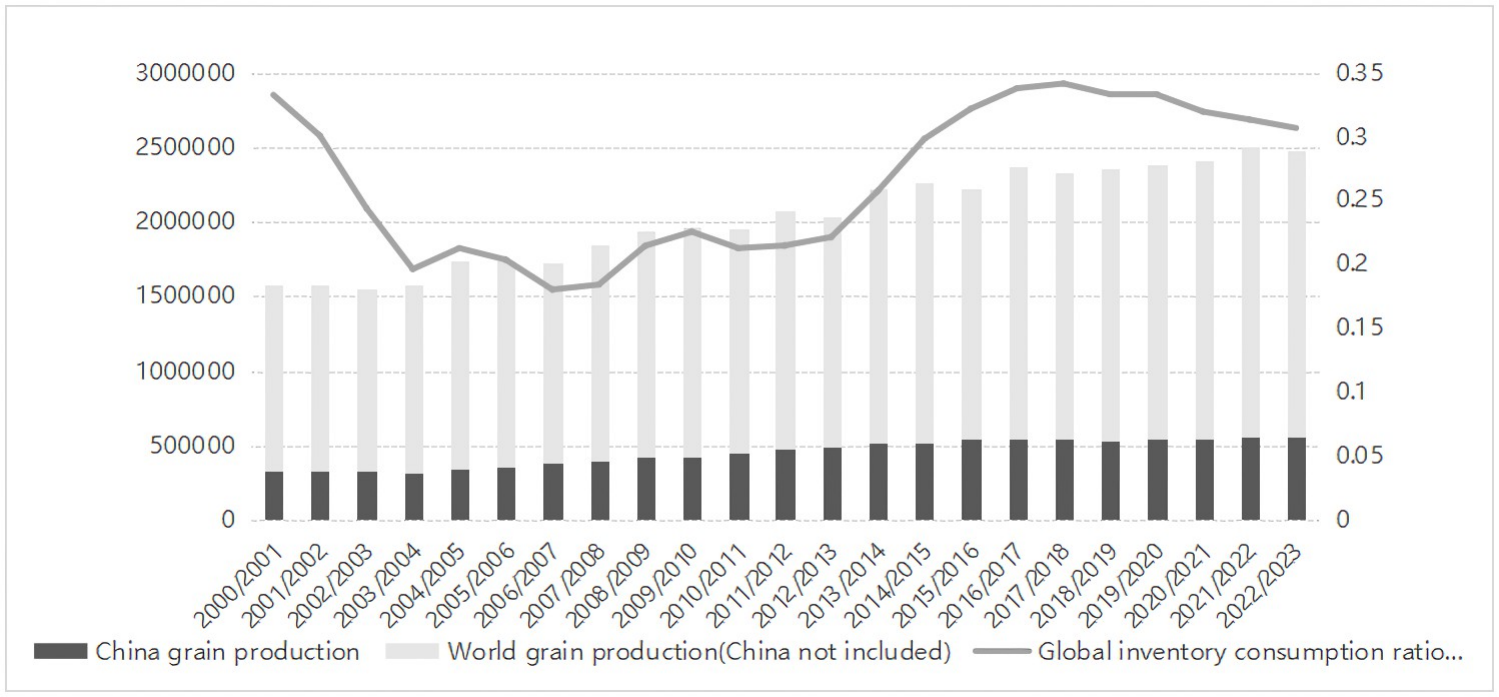
Changes in global food production and inventory.

Global food security is not a problem of total quantity of supply or demand, but rather an issue of uneven regional distribution. In terms of total quantity, global food supply is at a historically good level, and global food production during pandemic is continuing to increase, with production at a historical high point. As shown in Figs 9 and 10, global food production often rises during periods of rapid price growth. This means that there is enormous potential for global food production to increase. According to USDA data, in the 2022/2023 market year, global food production is expected to reach 2.76 billion tons, with a stock-to-use ratio as high as 30.7%, far above the FAO’s safety warning line. Although there is an abundance of food in terms of total quantity, the problem of regional imbalances in food supply and demand, coupled with the rapid decline in the global stock-to-use ratio, has led to the continuous deterioration of global food security. The global food supply and demand imbalance is characterized by the concentration of food production and export in the Americas and Black Sea regions, while import and consumption are dispersed. This pattern leads to many developing countries becoming heavily dependent on food imports. Furthermore, global food trade relies heavily on a small number of critical nodes, such as the export infrastructure in major crop-producing areas as well as narrow sea transportation points, such as straits and canals. Following the outbreak of the pandemic, coupled with international events like the Russia-Ukraine conflict, transport was disrupted leading to a rise in global food prices, putting some countries that heavily rely on food imports at risk. The number of hungry people worldwide continues to increase, according to FAO report, 193 million people in 53 countries or regions were facing severe hunger in 2021, an increase of 40 million people compared to 2020 [4].

### Monetary policy and food prices

Excessive US dollar liquidity has led to severe global inflation. Due to the impact of the COVID-19 pandemic, many developed countries have increased their currency issuance to stabilize their domestic economies. In 2021, the growth rate of the US M2 money supply reached as high as 19.1%, which is four times higher than that of 2019. The global currency oversupply has led to aggravated inflation worldwide. While the US CPI remained within 3% from 2017 to 2019, it has been increasing on a monthly basis since 2021, reaching as high as 8.6% in May 2022. The global commodity price index has increased by as much as 187.1% from its low point in 2020 to its high point during pandemic, while the global food price index has increased by 70.7%. Despite the tapering and interest rate hikes of the US dollar, the situation of excessive US dollar liquidity has not fundamentally changed. Therefore, excessive US dollar liquidity has been a major factor driving the rise in food prices during pandemic. In addition, financialization of food prices has also been an important factor causing the surge in food prices. International financial capital and multinational food traders collude to speculate on food shortage expectations by using international instability factors to amplify food price fluctuations, resulting in long-term deviations of food prices from the fundamental factors of supply and demand and cost [40].

### Intersection of climate change and geopolitical conflict

Extreme climate change and the Russian-Ukrainian conflict have increased the risk of global food production reduction, and the expectation of global food shortage has been strengthened, pushing up food prices. Since the outbreak of the Russian-Ukrainian conflict, the main driving force behind the global increase in grain prices has changed. Before March 2022, excessive US dollar liquidity and market panic caused by the pandemic were the main reasons for the global increase in grain prices. However, after 2022, geopolitical conflicts have been identified as the primary cause of the latest surge in global food insecurity. The Russian-Ukrainian conflict has had a profound impact on the global agricultural supply chain. Crude oil and fertilizers have gradually become the main driving force behind the rise in food prices. The surge in crude oil prices has pushed up transportation costs, while extreme climate change has led to expectations of reduced production in the world’s major production areas. The conflict has affected one-third of the world’s grain exports, and the expected grain production in Ukraine, known as Europe’s “granary,” has decreased by 50%(Data source: https://news.ifeng.com/c/8EyzDxIMrlX). The suspension of exports following the Russian-Ukrainian conflict has further worsened hunger in countries heavily reliant on wheat imports, including Afghanistan, Ethiopia, and Syria. The sustained rise in wheat prices has also ensnared Egypt, the world’s largest wheat importer, in the global food crisis vortex. According to World Food Programme(WFP), 60%of the world’s food-insecure population resides in areas affected by the Russian-Ukrainian conflict. Ukraine itself has experienced displacement and loss of livelihoods at an alarming rate.

### Export bans and global food insecurity

Export bans have exacerbated the expectation of global food shortages. Since the outbreak of the pandemic, global wheat and corn prices have continued to rise, triggering the strengthening of trade protection policies in various countries. Similar to the food crisis of 2007–2008, many countries around the world have introduced export bans on agricultural products to alleviate domestic food price increases and supply shortages. According to IFPRI statistics, from March to May 2022, 23 countries including India have successively introduced export bans on agricultural products such as wheat, corn, flour, tomatoes, vegetable oil, and beans. According to FAO data, restricted agricultural product exports account for 17% of global agricultural trade, and export bans have exacerbated the instability of the food supply chain. If Russia and Ukraine were to ban wheat exports as a result of their conflict, over 50 countries would encounter issues, with over 30% of their wheat supply affected. The majority of these countries are located in Asia and Africa and are classified as least developed or low-income nations(Data source: https://www.fao.org/3/nj164en/nj164en.pdf). Russia has also introduced export bans on fertilizers and other agricultural supplies. As the world’s largest producer of fertilizers, if the supply of fertilizers is severely disrupted in Russia, it may lead to a large-scale reduction in global food production.

### Interplay between energy and agriculture

The link between the food and energy markets has become stronger. Since the outbreak of the pandemic, international crude oil and natural gas prices have risen sharply, pushing up international food prices. Energy prices mainly affect food prices through three channels. Firstly, agricultural production is an energy-intensive industry, and the production of agricultural machinery and fertilizers highly depends on crude oil and other energy sources. Higher energy prices quickly translate into production costs. Since the outbreak of the pandemic, the prices of global fertilizers and crude oil have increased significantly, with fertilizer prices rising nearly 200% compared to 2019. Secondly, transportation costs affect food prices. Agricultural product trade highly depends on international transportation. With a global food production of 2.79 billion tons, nearly 17.9% of the food needs to be traded between countries. FAO data shows that since the outbreak of the pandemic, international freight rates have nearly tripled, leading to a rapid increase in international food prices. Thirdly, rising oil prices have pushed up the demand for biofuels, increasing the industrial demand for crops such as corn, resulting in a competition between food and fuel. Biofuels further deepen the link between high oil prices and high food prices. The proportion of important agricultural products such as food used for biofuel production is increasing, such as 30% of the corn production in the United States being used for the production of ethanol. When oil prices remain above $60, converting corn into biofuels will achieve breakeven. With oil prices remaining high, the drive for increased biofuel production will significantly exacerbate global food security.

Overall, in the future period, the multiple overlapping factors of geopolitical conflicts, extreme climate change, the industrialization and financialization of food and energy, and export restrictions may further worsen the global food crisis.

### Role of trade imbalances

The global food import and export market structure is imbalanced. International trade plays an increasingly important role in maintaining global food security. Most grain-deficient countries depend heavily on imports from the international market, while a few major exporting countries have a large impact on the international market, and the trade measures of large grain exporting countries pose a greater risk to global food security. According to FAO, from the perspective of the proportion of global grain trade volume and consumption, it has reached 17.4% in 2020, and will slightly decrease to around 17% by 2022; in terms of varieties, rice, coarse grains, wheat and soybean trade volume and consumption proportions are about 10.2%, 15.1%, 25%, and 44.2%, respectively. Import trade plays a crucial role in safeguarding global food security, avoiding local food crises and spill-over effects, especially for soybeans and wheat with high trade consumption ratios. The distribution of global food and fertilizers import and export markets has been uneven for a long time, with a high concentration of exports and relatively scattered imports. According to data from the USDA, the combined export volume of the top five rice, wheat, corn, and soybean exporting countries in 2021 accounted for as high as 78.9%, 66.3%, 88.5%, and 90.6% of the world’s total exports, respectively. The export volume of the top three global fertilizer exporting countries accounts for about 70%. In terms of grain importing countries, rice and wheat and other staple food imports are mainly concentrated in Asian countries with large populations and scarce land resources as well as in African countries with underdeveloped agricultural production. Feed grain imports, such as corn and soybeans, are concentrated in East Asia and Europe, where there is a greater demand for livestock farming. The global food supply chain still experiences instability, and key points in the smooth flow of global food logistics are affected by geopolitical factors, which will continue to affect global food security.

## Conclusions and policy recommendations

This study employs the CatBoost machine learning model to analyze the relative significance of influential factors, utilizing international food price data spanning from January 2000 to May 2022. The study identifies the most significant factors affecting the global rise during pandemic in grain prices. The results show that the primary factors are the supply of the US dollar and the price of crude oil. The unlimited quantitative easing monetary policy of the US dollar is the main driver of the global rise during pandemic in grain prices. The main impact of crude oil on grain prices is cost-push, including the cost of transportation, which has increased the intermediate cost. The study also found that the supply and demand shocks are another significant factor affecting the rise in grain prices. The complex international situation has amplified the expectations of crop reduction and demand growth, further exacerbating the global food crisis. The study suggests that the global food security issue is not a total quantity problem, but rather a problem of regional distribution. Extreme climate change and the Russo-Ukrainian conflict have increased the risks of global food production declines, and the expected increase in global food demand has further exacerbated the problem. In the context of the global food scarcity expectations, the impact of crop reduction and instability expectations on grain prices will increase further. Other factors, such as the economic policy uncertainty caused by the Russo-Ukrainian conflict, also have an increasingly significant impact on global food prices. Thus, we need to pay attention to the possible risks of the Russo-Ukrainian conflict and other international uncertainties, which may change global agricultural product trade structure and the potential risks faced by the agricultural product import supply chain, as well as the sustained high international food prices, which may intensify domestic inflation risks.

Based on the above research conclusions, this study proposes several recommendations. Firstly, developed countries should take responsibility for providing public goods to ensure the smooth and stable operation of the global agricultural supply chain, guaranteeing the stability and openness of the global food supply chain. Secondly, due to the unequal distribution of resources, grain trade is indispensable to global food security. Therefore, we should actively promote the establishment of more reasonable global trade rules and provide fair and reasonable international environments for developing countries. Thirdly, countries should restrict the production of biofuels and reduce non-food demand for grain. Finally, to mitigate the negative impact of soaring food prices on food security, it is crucial to promote the transformation of the agricultural food system towards greater sustainability and resilience. This transformation should involve halting the expansion of agricultural food production into natural habitats, fostering interdisciplinary cooperation to achieve harmonious and healthy development among people, animals, and the environment, and stabilizing macroeconomic policies and creating buffers between them and the agricultural food system.

Admittedly, there are several limitations to the study that leave room for future research. One of the limitations is that the study only uses macroeconomic monthly data, which may not capture the nuances of the food market. Future research could consider using more granular data to improve the accuracy of the analysis. The significance of the variables might vary beyond the data range spanning January 2000 to May 2022, yet the methodology introduced in this paper remains applicable. Another limitation is that the study did not use deep learning models such as Informer, Recurrent Neural Network(RNN) model or Long Short-Term Memory(LSTM) model, which may perform better on time-series data. Future studies could explore the use of more advanced deep learning models to improve the accuracy of the analysis.

Moreover, the study only focuses on variables such as oil prices and the US dollar supply, and does not consider other potential factors that could influence food prices, such as climate change or supply chain disruptions. Future research could investigate the impact of climate change on food prices and food security and explore the potential risks arising from international uncertainty factors, such as geopolitical conflicts or export restrictions.

## Supporting information

S1 Dataset (XLSX)
